# Adaptation in Sound Localization Processing Induced by Interaural Time Difference in Amplitude Envelope at High Frequencies

**DOI:** 10.1371/journal.pone.0041328

**Published:** 2012-07-27

**Authors:** Takayuki Kawashima, Takao Sato

**Affiliations:** 1 Department of Cognitive and Behavioral Sciences, Graduate School of Arts and Sciences, University of Tokyo, Tokyo, Japan; 2 Department of Psychology, Graduate School of Humanities and Sociology, University of Tokyo, Tokyo, Japan; University of Salamanca- Institute for Neuroscience of Castille and Leon and Medical School, Spain

## Abstract

**Background:**

When a second sound follows a long first sound, its location appears to be perceived away from the first one (the localization/lateralization aftereffect). This aftereffect has often been considered to reflect an efficient neural coding of sound locations in the auditory system. To understand determinants of the localization aftereffect, the current study examined whether it is induced by an interaural temporal difference (ITD) in the amplitude envelope of high frequency transposed tones (over 2 kHz), which is known to function as a sound localization cue.

**Methodology/Principal Findings:**

In Experiment 1, participants were required to adjust the position of a pointer to the perceived location of test stimuli before and after adaptation. Test and adapter stimuli were amplitude modulated (AM) sounds presented at high frequencies and their positional differences were manipulated solely by the envelope ITD. Results showed that the adapter's ITD systematically affected the perceived position of test sounds to the directions expected from the localization/lateralization aftereffect when the adapter was presented at ±600 µs ITD; a corresponding significant effect was not observed for a 0 µs ITD adapter. In Experiment 2, the observed adapter effect was confirmed using a forced-choice task. It was also found that adaptation to the AM sounds at high frequencies did not significantly change the perceived position of pure-tone test stimuli in the low frequency region (128 and 256 Hz).

**Conclusions/Significance:**

The findings in the current study indicate that ITD in the envelope at high frequencies induces the localization aftereffect. This suggests that ITD in the high frequency region is involved in adaptive plasticity of auditory localization processing.

## Introduction

Experience-dependency of auditory spatial processing has attracted the attention of many researchers [1]–[5]. One example of the experience-dependent plasticity of the sound localization system is the localization/lateralization aftereffect. This is a perceptual phenomenon where, in typical settings, after a long exposure to a sound (from seconds to several minutes), sounds tend to be perceived away from the adapting sound [3], [5]–[7].

The localization aftereffect has often been thought to reflect an efficient neural coding of the sound localization system that takes prior context into account (the prior context means the history of stimulus). In one study using adult ferrets [3], responses of neurons in the inferior colliculus that are sensitive to interaural level difference (ILD), an important localization cue, were investigated. The adapters were 5-s noise sequences in which ILD rapidly fluctuated according to a Gaussian distribution. It was found that, in single unit recordings, the positions of rate-ILD functions of neurons shifted toward adapters' mean ILD after adaptation, while the slopes of the functions were almost constant when variance of ILD distributions of the adapters was not changed. These tendencies suggest that the sound localization system efficiently utilizes limited neural resources to code the positions of sounds by adapting to prior experiences. Moreover, the change in the sensitivity of the neurons could theoretically explain the localization aftereffect of human listeners. Other studies also concluded that the localization and lateralization aftereffect reflects the adaptive neural coding of the sound localization system [4], [8].

It is important to identify auditory localization cues that induce the aftereffect in sound localization, because this might determine how, or by which cue, the auditory system adapts to sound sources. Because multiple localization cues usually coexist in everyday listening situations, the roles or contribution of each cue in sound localization is typically not evident without specific investigation. Sound localization depends on a variety of cues, such as interaural temporal and level differences (the former is abbreviated as ITD), and monaural spectral cues. There are two types of ITDs as localization cues [9], [10], the first one being interaural difference in the fine temporal structure of the sound waves, that presumably only affects localization in the low-frequency region (below 2 kHz). The second type is an interaural temporal difference in the envelope that presumably affects localization in the high-frequency region. Three studies have suggested that ITD in temporal fine structure may contribute to the aftereffect [5]–[7]. Thurlow and Jack, by using bandpass noise (from 1 kHz to 3 kHz), showed that an ITD in the adapting sound affects the lateralization aftereffect's magnitude. Others reported a similar tendency using pure-tones (below 800 Hz) as stimuli. Because in these studies they used low frequency ITD or pure-tones as stimuli, it appears that fine structure ITD contributes to the aftereffect. No study to date, however, has explicitly demonstrated any contribution from ITD in the envelope. The main purpose of the present study is to examine whether ITD in the envelope might induce the localization (lateralization) aftereffect.

In sound localization and lateralization it has been repeatedly reported that ITD, when conveyed by high-frequency stimuli (i.e., ITD in the envelope), is a weaker cue than ITD that is conveyed by low-frequency stimuli [9]–[11]. For example, for a given ITD, sound images produced by (narrow-band) high-frequency stimuli, like sinusoidally amplitude modulated tones (SAM tones), are perceived to be much closer to a listener's midline than sound images produced by comparable low-frequency stimuli (e.g., pure tones) [12]. Logically, this tendency suggests, at the least that there are differences between the (central) binaural mechanisms that mediate interaural interactions in low- and high-frequency regions, respectively.

Recent research, however, has indicated that the differences in ITD sensitivity between low- and high-frequency regions might result from monaural, that is neural peripheral, processing prior to binaural interaction. In other words, it has supported the view that the binaural mechanism that processes the two types of ITD (envelope and fine-structure ITDs) operates uniformly across frequency [13]. In this view, the differences in the sensitivity due to frequency are observed because the (monaural) temporal information that is provided by high-frequency stimuli to this binaural mechanism is less effective than the information provided by the low-frequency stimuli. Evidence supporting this view has come from experiments using *transposed* stimuli. Transposed stimuli represent a particular type of high-frequency stimulus that is devised to provide the binaural localization system with temporal information that is identical to that produced by comparable low-frequency stimuli. Typically, these stimuli have been created by modulating a high-frequency carrier with a signal that is a low-pass filtered and half-wave rectified version of the comparable low-frequency stimulus [14]. Recent localization experiments have shown that this type of (envelope) ITD, which is conveyed by the transposed stimuli, is much more effective than ITD conveyed by traditional high-frequency stimuli, SAM tones; furthermore, it is almost as effective as ITD conveyed by the low-frequency stimuli [15]–[17]. In sum, findings using transposed stimuli indicate that the binaural mechanism that processes ITD operates uniformly across frequency [14]–[17].

A problem which has not been addressed concerns the adaptivity of the binaural mechanism itself. In two experiments, the present study directly addresses the question: Is the localization mechanism adaptive to ITD in the envelope, as was reported to be to ITD in the fine-structure? Experiment 1 investigated whether ITD in the envelope induces the lateralization aftereffect, using high frequency transposed stimuli. Experiment 2 examined selectivity of the adaptation observed in Experiment 1, introducing spectral differences between adapter and test sounds. Additionally, listeners were tested in a psychoacoustic task that differed from that used in Experiment 1 to confirm robustness of this phenomenon. Obtained data showed ITD in the envelope at high frequencies elicited the localization aftereffect.

## Experiment 1

### Ethics Statement

The research protocol for the current study was approved by Research Ethics Committee in the University of Tokyo, graduate school of arts and sciences. Informed written consent was obtained from all participants.

### Materials and methods

#### Participants

Five adults (four males and one female) participated in Experiment 1 (referred to as S1–S5). They ranged from 22 to 29 years old. A tone audiogram (125 Hz to 8 kHz) confirmed that they each had normal hearing ability (below 20 dBHL). Four of the participants had already participated in lateralization experiments several times The rest (S2) had ample experience in psychoacoustic experiments, but had no experience of lateralization. All participants underwent two practice sessions before data collection. S4 was the first author.

#### Apparatus and Stimuli

Sound signals were generated digitally (sampling frequency of 44.1 kHz and quantization of 16 bits) by a personal computer (Macintosh G4). They were led to a D/A converter (Stax DAC Talent) and presented through headphones (Sennheiser HDA200). The experiment was conducted in a sound attenuated room.

AM tones were used for both adapter and test stimuli. The carrier in each case was a 4.5-kHz sinusoid. The modulating signal (modulator) was a half-wave rectified and low-pass filtered tone with a frequency of 128 Hz. The low-pass filter had a cutoff frequency of 700 Hz. The generated stimuli had a power spectral peak at 4.5 kHz and side bands at ±128, ±256, ±512 Hz, around the peak. Although many studies on envelope ITD have used sinusoidally amplitude modulated (SAM) tones as stimuli [18], we used the transposed stimuli (see Introduction) because they have been reported to be lateralized more easily than the SAM tones and have been reported to provide the binaural localization system with temporal information that is identical to that produced by comparable low-frequency stimuli [15]–[17]. Stimuli were presented at 60 dBSPL. An artificial ear (Brüel & Kjær Type 4153) and a microphone (Brüel & Kjær Type 4134) were used to calibrate the level. The difference in level between the peak and the components below 2 kHz was more than 60 dB, hence the stimuli had no significant energy within audible range below 2 kHz. The phases of the carrier waves were the same across the left and the right channels, and the ITDs were introduced by shifting the phase of the modulator. Both the left and the right channel modulators were gated simultaneously.

Before each block started, six adapter signals, each of 30 s duration, were digitally generated. They were presented in succession with minimal intervals between them and added up to approximately 180 s of adaptation period. In addition, a short adapter (5 s) was presented before each trial that was sampled from the six longer signals to reinforce the adaptation. The interval to be sampled was randomly determined for each trial. The method for presenting adapters (i.e., repeated presentation of the adapters before each block and each trial) followed that of previous studies [7], [19]. The starting phase of the carrier and the modulator was randomly determined from a uniform probability distribution. A new test sound was generated for each trial.

The test sound lasted 0.8 s with a 0.9 s silent interval between the adapter and the test. These temporal intervals were adopted because a pilot study had shown that it was easy for the participants to locate the positions of the test stimuli under these settings. In the no-adapter condition, there was no time interval corresponding to the adapter presentation. Raised-cosine ramps (0.06 s) were added to the onsets and the offsets of stimuli.

#### Procedure

In the experiment, the participants reported their perceived position of the test stimulus by moving a pointer to the corresponding place on a circle displayed on a computer display. They were told to regard the circle as their heads. The pointer was a vertical line that moved only in a horizontal direction. In every trial, the initial position of the pointer was randomized. The position of the pointer was recorded in 485 units. In the data analysis, the recorded value was normalized linearly by designating the left edge, the center, and the right edge of the circle as minus one, zero, and plus one respectively.

In the adaptation sessions, the adapter had one of three ITDs during the adapter presentation periods (180 s before each block and 5 s before each trial): −600, 0, or 600 µs (Negative values indicate that the left channel leads relative to the right). The participants were instructed to listen passively to the adapter and attend to written words (“Long sound”) that were presented at a fixed position of the computer display. For the test stimuli, the ITD was varied in 13 steps: ±1500, ±1200, ±900, ±600, ±400, ±200, and 0 µs. ILD was fixed at 0 dB throughout the experiment. In the condition where no adapter was presented, participants responded 36 times to each ITD value of the test. In the condition where the adapter was presented, three of the five participants responded 18 times to each test ITD value (S2, S3, S5) and the rest responded 24 times (S1, S4). Each test ITD value was repeated three times per block, hence 39 trials in each block. Each session started with two control blocks followed by three or four blocks of the adapting condition. The adapter ITD remained constant throughout each session and only one session was conducted a day. Sessions were conducted in random order the first time around, and then the three ITD conditions were repeated again in a new order. A short rest period was provided between blocks. Each session took about 1 h.

During the experiment, the low-pass filtered noise was presented with the adapter and the test sound to prevent participants from using ITD information conveyed by any, possible low-frequency distortion-products that results from non-linearity [10], [18]. The filter had a cutoff frequency of 1.2 kHz and the noise was binaurally uncorrelated. The filtered noise had a level of 63 dBSPL, and the noise started 1.2 s before onsets of the adapter or the test sound, and stopped 1.2 s after offsets.

### Results and discussion

**Figure 1:**
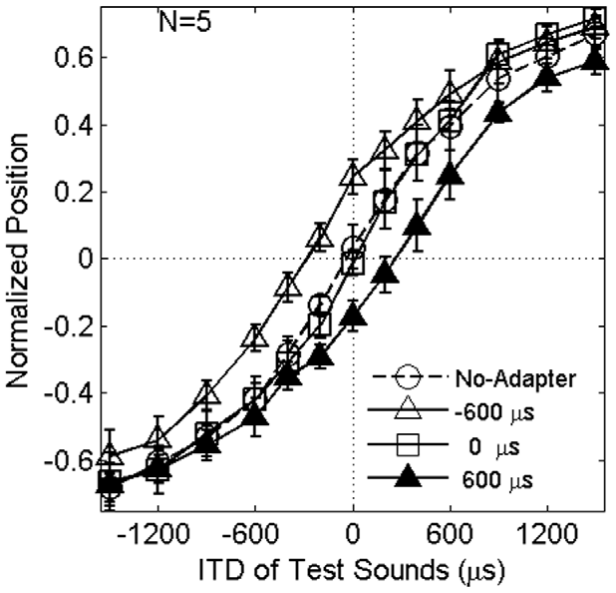
Average normalized responses as a function of the ITD of the test stimulus. Error bars represent standard errors. After the adaptation at ±600 µs, the subjective midline shifted towards the position of the adapter. Error bars show SEs.

Psychometric functions for each participant were drawn from the average responses to each of the four adapter conditions. The curves for means across the participants are shown in Figure 1. When the adapter's ITD was 0 µs (represented by unfilled squares), the average responses were not largely different from those in the no-adapter condition (represented by unfilled circles).

On the other hand, the average responses deviated from the no-adapter condition when the magnitude of the adapter's ITD was −600 and 600 µs (represented by unfilled and filled triangles in Figure 1, respectively). In these conditions, the subjective midline derived from psychometric functions shifted towards the adapter's position. Similar shifts were reported in studies of localization aftereffects where low-frequency pure-tones [7] and band-limited noise that contained energy in the low-frequency region [6] were used. A one-factor ANOVA and the following Tukey's Honestly Significant Difference (HSD) Tests for ITDs corresponding to the subjective midline (estimated by linear interpolation) showed that an effect of types of adapters was significant F(3, 12) = 14.8, p<.001 and that there was no significant difference between the 0 µs and the no-adapter condition (p>0.1). There were significant differences between the ±600 µs and the no-adapter condition (p<0.05 in both cases). The present results indicate that ITD in the envelope is one of the localization cues that induce the lateralization aftereffect.

To evaluate the slope of the functions, normal ogives were fitted to each participant's data and the estimated indexes (i.e., sigma) were submitted to a one-factor ANOVA. Effect of the adapter conditions was not significant, F(3,12) = 0.66, p = .59, partial η^2^ = 0.14, indicating that the four adapter conditions did not differ statistically in the slope of these functions.

The data in Figure 1 indicate that perceived positions for the test stimuli presented at the same position as the adapters (i.e., presented with same ITD values as those of the adapters) seemed to shift toward the midline. A similar tendency has been reported when adaptation to broad-band noise, presented at 30° from the midline, was examined in free-field [19]. Re-analysis of the data using normalized perceived positions was conducted: A two-factor ANOVA (4 adaptation conditions ×13 test ITD conditions) revealed a significant effect of interaction, F(36, 144) = 2.80, p<.001, partial η^2^ = 0.41. Among the related test stimuli, results in one condition (–600 µs ITD test) showed significant shift toward the midline when the adapter was presented with –600 µs ITD (p<.05, in a HSD test), and results in the other test conditions did not reach significance (p>.1). It was not clear that the observed shift reflected a systematic effect of the adaptation.

The results of Experiment 1 suggest that ITD in the envelope contributes to the lateralization aftereffect. It is not clear, however, whether the observed effect reflects an adaptation during perceptual processing or is a result of a response bias to the adapter at the cognitive processing level. In the next experiment, the effect of the adapter was examined using a different spectrum for the adapter and test. The selectivity of adaptation should enable us to distinguish between cognitive and perceptual effects [20]. Moreover, the second experiment employed a different task from that used in Experiment 1 (i.e., a two-alternative forced choice (2AFC) task). The 2AFC task was useful to test the robustness of the perceptual change observed in Experiment 1, and, in addition, it had been used in previous adaptation studies [7], [8].

## Experiment 2

### Materials and methods

#### Participants

Three males participated in Experiment 2 (referred to as S3, S6, and S7). They ranged from 22 to 31 years old. An audiogram confirmed their normal hearing ability. Only S3 had participated in Experiment 1. All had experience in lateralization experiments and were given more than three practice sessions before data were collected.

#### Stimuli

As well as using almost identical AM tones to those in Experiment 1, low-frequency tones were also used in this experiment. The AM sounds were generated in the same way as they had been in the first experiment except that the carrier frequency was changed from 4500 Hz to 4043 Hz, and there were two values of the modulation frequency: 128 and 256 Hz, and the low-frequency tones also had frequencies of 128 and 256 Hz. A different(lower) carrier frequency from Experiment 1 was used. We wished to measure magnitude of the aftereffect using the lower carrier frequency (than in Experiment 1) in order to prepare for another experiment (not reported here). The stimuli were presented at 54 dBSPL. For the AM stimuli, there was a 60 dB difference between the carrier component and the components below 1.9 kHz, hence the stimuli had no audible energy below 1.9 kHz. There was a 120 s initial adaptation at the beginning of each block, and a short adapter (2 s) was presented before each trial. The duration of the adapters were reduced from 180 s and 5 s in Experiment 1 to collect data efficiently.

#### Procedure

The procedure was similar to that of Experiment 1, except for the following: The participants' task was to judge whether the test stimulus was to the left or to the right relative to the midline (two-alternative forced choice). There were two types of adapters in their frequency contents: one consisting of an AM sound whose modulation frequency was 128 Hz and another with a tone burst of 128 Hz. The adapters had an ITD of −937 or 937 µs. Thus, there were four conditions of adapters. There were four types of test stimuli: two consisted of AM tones with modulation frequencies of 128 and 256 Hz (referred to as AM128 and AM256, respectively), and the other two consisted of pure-tones of 128 and 256 Hz (referred to as P128 and P256). The ITD was varied between −625 and 625 µs in nine steps when the test stimulus was AM128 and P128. It was varied between −312 and 312 µs when the test stimulus was P256. The variable range was either between −390 and 390 µs (S3, S7) or between −468 and 468 µs (S6) in the AM256 condition. The range in the AM256 condition was determined for each participant for an efficient data collection. Thus, there were 36 conditions in the test stimulus for each participant (four sound types × nine ITD values). In the no-adapter condition, the participants responded at least 24 times for each test condition. In the adaptation session, one of them responded 16 times (S6) and the rest responded 24 times for each test condition. Each test stimulus was repeated twice or three times in random order in a block. When a session started, one block of the control condition was conducted first, followed by four blocks with adapters. The four adapters were presented in different sessions and in random order across the participants. Then the series of adapters was repeated twice (S6) or three times (S3 and S7) in randomized orders. The low-pass filtered noise was not presented in this experiment because the noise itself might have an unpredictable effect especially on the lateralization of the low-frequency tones [7].

### Results and discussion

**Figure 2:**
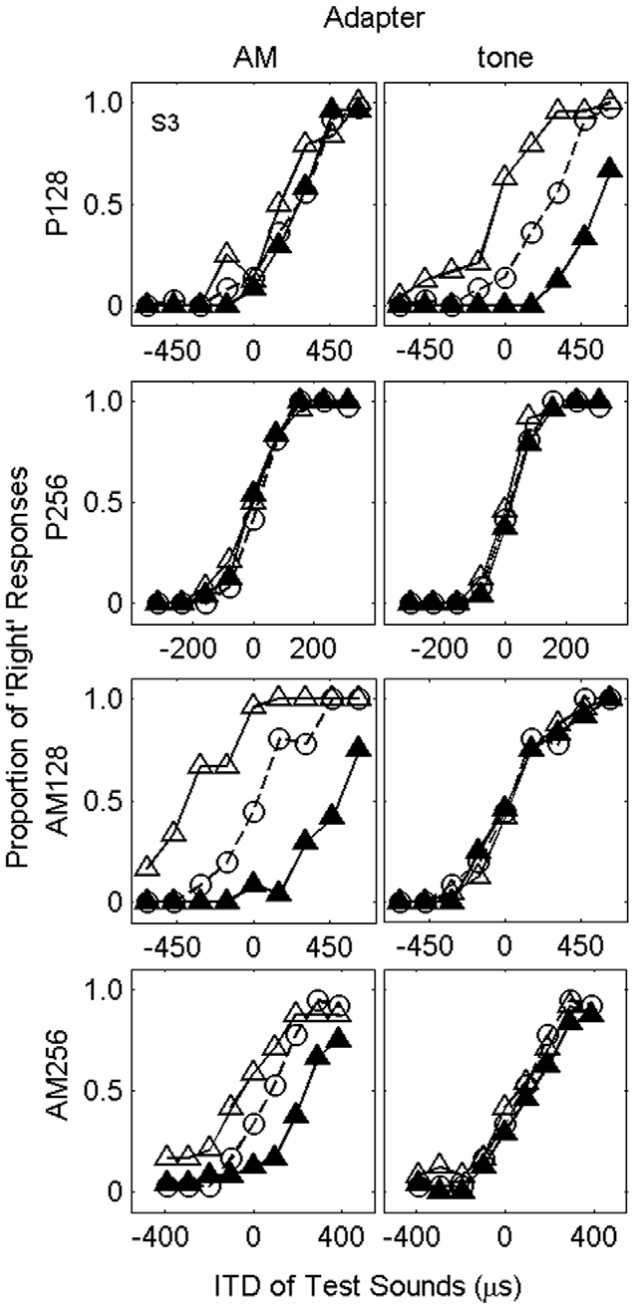
Proportion of “right” responses as a function of the ITD of the test stimuli of one participant (S3). The left panels show the results in AM adapter condition (128-Hz modulation frequency), and the right panels show those in tone adapter condition (128 Hz). Each row displays results in each test condition: The abbreviations P128 and P256 refer to the 128- and 256-Hz tone respectively. AM128 and AM256 refer to the AM sounds that were modulated with 128 and 256 Hz. Unfilled circles indicate the results in the no-adapter condition. Filled and unfilled triangles indicate the results in the −937 and 937 µs ITD conditions respectively. There is a relatively larger aftereffect when both the adapting and test stimuli are AM tones.

Psychometric functions were drawn from the proportion of “right” responses to each ITD in the test stimulus. In Figure 2, the psychometric functions of one participant (S3) for AM adapters (left side panels) and tone adapters (right side panels) are shown. The results for AM tests, displayed in the third and fourth rows from the top, show that psychometric functions shifted towards the ITD of the adapter after adaptation (unfilled triangles represent the responses after adaptation to the left side adapter, while filled triangles represent those to the right side). This indicates that the subjective midline of the participant shifted towards the ITD of the adapter, as in Experiment 1. The results for tone tests, displayed in the first and second rows, show that the AM adapters did not affect the responses, however, the tone adapters of 128-Hz affected the responses for the tone tests. Similar tendencies of the aftereffect were observed for the remaining two participants (Figures S1 and S2).

**Figure 3:**
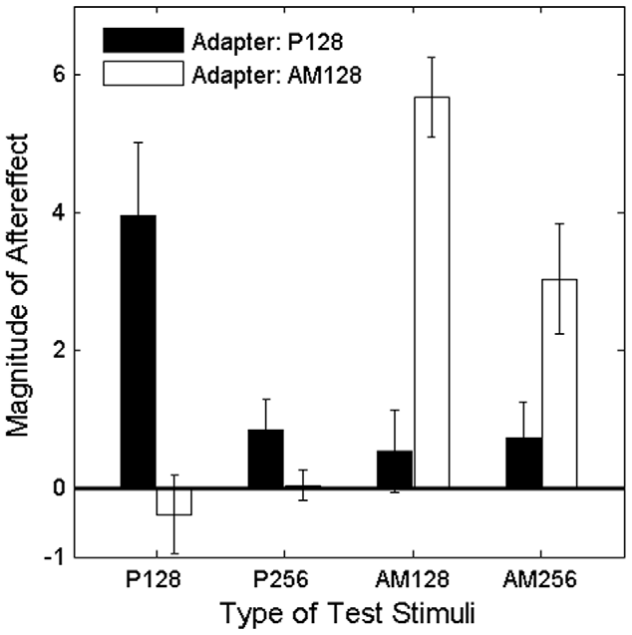
The average magnitude of the aftereffect in each adapter condition as a function of the type of test stimuli. See the caption of Figure 2 for explanation of abbreviations such as P128. There are relatively large aftereffects when the adapter and test stimuli are of the same type. Error bars show SEs.

To quantify the magnitude of the aftereffect, the proportions of right responses obtained when the adapter was presented at the left side were subtracted from those when the adapter was presented at the right side, and then they were summed across ITD conditions of the test stimulus [20]. Larger, positive values indicate a stronger localization aftereffect. To quantify the magnitude of an aftereffect in Experiment 2, the method was modified from that of Experiment 1 in order to avoid extrapolation of a psychometric function of a listener (S6) in estimating a point of subjective midline. Resulting values averaged across the participants are plotted in Figure 3. When the adapters were AM sounds, relatively large aftereffect was observed (two rightmost white bars). To the contrary, responses for pure tone tests (P128 and P256) did not exhibit a significant change regardless of the envelope ITD (two leftmost white bars). A 2×4 ANOVA (2 types of the adapter ×4 types of the tests) showed a significant interaction, F(3,6) = 47.5, p<.001, partial η^2^ = 0.96; following up on this, in the AM adapter condition, the simple main effect of the test types was significant, F(3,12) = 44.9, p<.001, η^2^ = 0.92. Differences between AM128 and both of the two tone tests were significant in the subsequent HSD tests (p<.05 in both cases). These analyses indicate that adaptation to ITD in the envelope at the high frequency region did not affect the lateralization of tones at the low frequency region. These findings have two implications: First, they indicate that apparent changes of listeners' responses after adaptation reflect a real perceptual change. That is, if the adapter effect was caused by a criterion shift, then the AM adapter should have changed the position of all test sounds, including P128 and P256. Second, the selective adaptation effect suggests that a difference tone, with a frequency corresponding to the modulation frequency of the adapter (128 Hz), if produced internally by a non-linearity of the inner ear, did not play a significant role in this aftereffect. If an internally produced 128 Hz difference tone had been present, it would have affected the localization of tests because the adapter was presented for relatively long time.

When the adapting stimulus consisted of a 128-Hz tone (black bars in the figure), the aftereffect was observed for the 128-Hz tone test stimulus (a leftmost bar), but the effect was not consistently observed in the other test conditions. The studies of the lateralization aftereffect for low-frequency tones using lateralization and discrimination tasks have shown that the aftereffect is frequency selective [7], [8]. This tendency is replicated in the current study. The observed magnitude of the aftereffect in this condition was comparable for the magnitude in AM128 for AM adapters. The possibility has been reported that the essentially same mechanism in localization processing works for detecting both of the two types of ITDs: fine structure ITD at low frequencies and envelope ITD at high frequencies [13]. The similarity of the results between the two types of ITD conditions is consistent with this possibility, although exact comparison for the validation seems to require further studies.

## Discussion

In Experiment 1, when the adapter had an ITD of 0 µs no effect was observed. Several studies, however, have reported aftereffects for adapters with 0 µs ITD [6], [7]. We could not find a clear reason for the lack of the effect, however, there are at least two possibilities. First, the effect of the adapter might not be evident because intervals of ITD between each of the test conditions were too wide to capture the aftereffect. The lateralization aftereffect was reported to be ITD selective (at least) in the low frequency region. For example, it has been reported that the aftereffect, induced by adapters having an ITD of 0 µs, is observed only in a restricted area of ITD, around 220 µs when 400 Hz tones were used as stimuli [7]. Therefore, it is possible that the ITD intervals between test sounds in Experiment 1, 200 µs or 300 µs, are too wide to detect changes caused by the adapters. Second, the lack of the effect may be a result of reduced sensitivity to amplitude modulation itself following adaptation. It has been reported that the threshold for detecting amplitude modulation increases following an exposure to SAM tones [21], [22]. It has also been reported that when the depth of modulation decreases and the amount of ITD remains constant, lateralization of SAM tones decreases [10], [18], [23]. In Experiment 1, it is likely that exposure to the adapter decreased the effective depth of modulation, and this led to a shift towards the midline in the perceived location of the test stimuli because the same amount of ITD lost its ability to lateralize the sound after adaptation. No effect of the adapter was observed, because this tendency and the localization aftereffect canceled each other out.

Recent studies on adaptation in sound localization have proposed that adaptation results from a modification of the output pattern of location-selective units during auditory processing [5], [8]. Those units are usually related to neurons that are selectively sensitive to ITD in the fine structure [7], [8] or ILD [2]. The aftereffect induced by an ITD in the envelope (Experiments 1, 2) can also be explained qualitatively via an almost identical scheme. According to this model, the perceived location of sounds is determined by the peak (or possibly other summary indexes) of the pattern created by the output of the location selective units. An exposure to the adapter reduces the output activity of certain units which corresponds to an adapter's location, thereby shifting the output distribution peak laterally. This then shifts the perceived location of sounds away from the adapter's location. The results of the present study are consistent with the model mentioned above. This interpretation is partly supported by Batra et al. who have reported that there are high-frequency neurons in the inferior colliculus of cats that are sensitive to ITD in the envelope [24].

### Concluding remarks

Several researchers have proposed that the auditory localization aftereffect results from neural adaptation of spatial processing to the prior stimulus context. It is important to identify localization cues that induce the aftereffect in sound localization, because this might reveal ways in which the auditory system adapts to sound sources in everyday listening situations, where multiple localization cues usually co-exist. In the current study, a possible effect of ITD in the envelope at high-frequency region was examined.

In Experiment 1, participants were required to adjust the position of a pointer to the perceived position of test stimuli under several adapting conditions. The results showed that ITD in the envelope of an AM adapter shifted the perceived position of the test sound away from this adapter and that this tendency was consistent with the localization aftereffects reported in earlier studies [3], [5]–[7]. In Experiment 2, the effect of the AM adapter was confirmed using a forced-choice task. It was found that the AM adapter did not change the perceived position of pure-tone tests at the low-frequency region. This frequency selectivity of the adaptation was interpreted to mean that the observed effect of the adapter was not a result of a general change of a listener's criterion after adaptation, but rather reflected a real perceptual change. Findings in the current study indicate that ITD in the envelope at the high frequency region induce the lateralization aftereffect and suggest that the envelope ITD contributes to adaptive aspects of auditory localization processing. Functional roles of neural adaptation in the sound localization system and its relation to the localization aftereffect have been studied mainly for ITD in the fine structure [4], [8] and ILD [2]. Neurophysiological study on the neural adaptation to ITD in the envelope would be useful in developing greater understanding of the adaptive nature of auditory localization processing.

## Supporting Information

Figure S1 **Proportion of “right” responses of each participant as a function of ITD of the test stimuli in the AM adapter condition (128-Hz modulation frequency).** Each row displays each participant's result. Each column displays the results in each type of the test stimulus. See the caption of Figure 2 for explanation of abbreviations such as P128.
(TIF)

Figure S2 **Proportion of “right” responses of each participant as a function of ITD of the test stimuli in the tone adapter condition (128-Hz).** See the caption of Figure S1 for details.
(TIF)
