# Supplementary figures and images for: Adaptation in Sound Localization Processing Induced by Interaural Time Difference in Amplitude Envelope at High Frequencies
Source: PLoS One. 2012 Jul 27;7(7):e41328. doi: 10.1371/journal.pone.0041328 (PMC3407190; doi:10.1371/journal.pone.0041328)

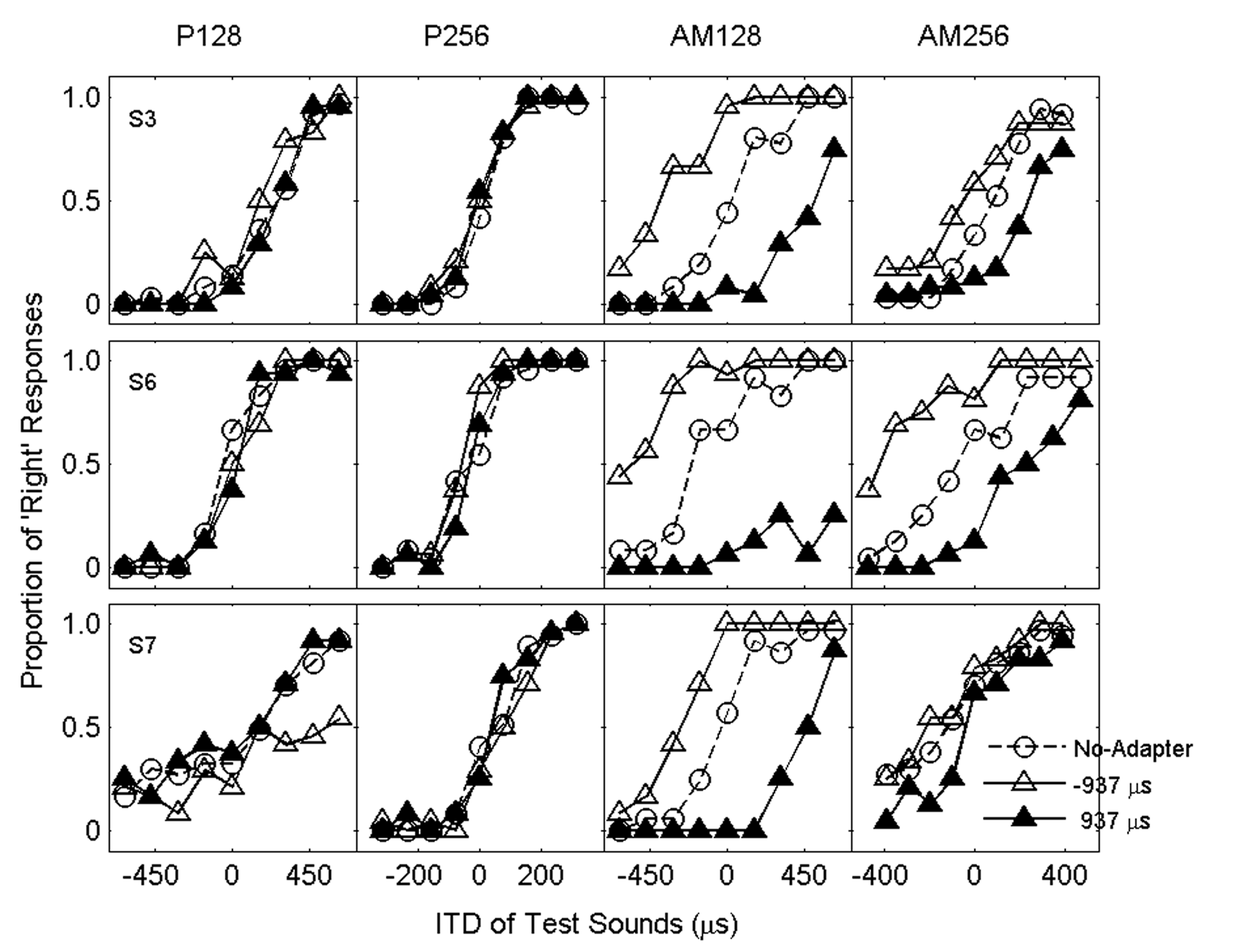

Supplement: Figure S1 — Proportion of “right” responses of each participant as a function of ITD of the test stimuli in the AM adapter condition (128-Hz modulation frequency). Each row displays each participant's result. Each column displays the results in each type of the test stimulus. See the caption of Figure 2 for explanation of abbreviations such as P128. (TIF) [file pone.0041328.s001.tif]

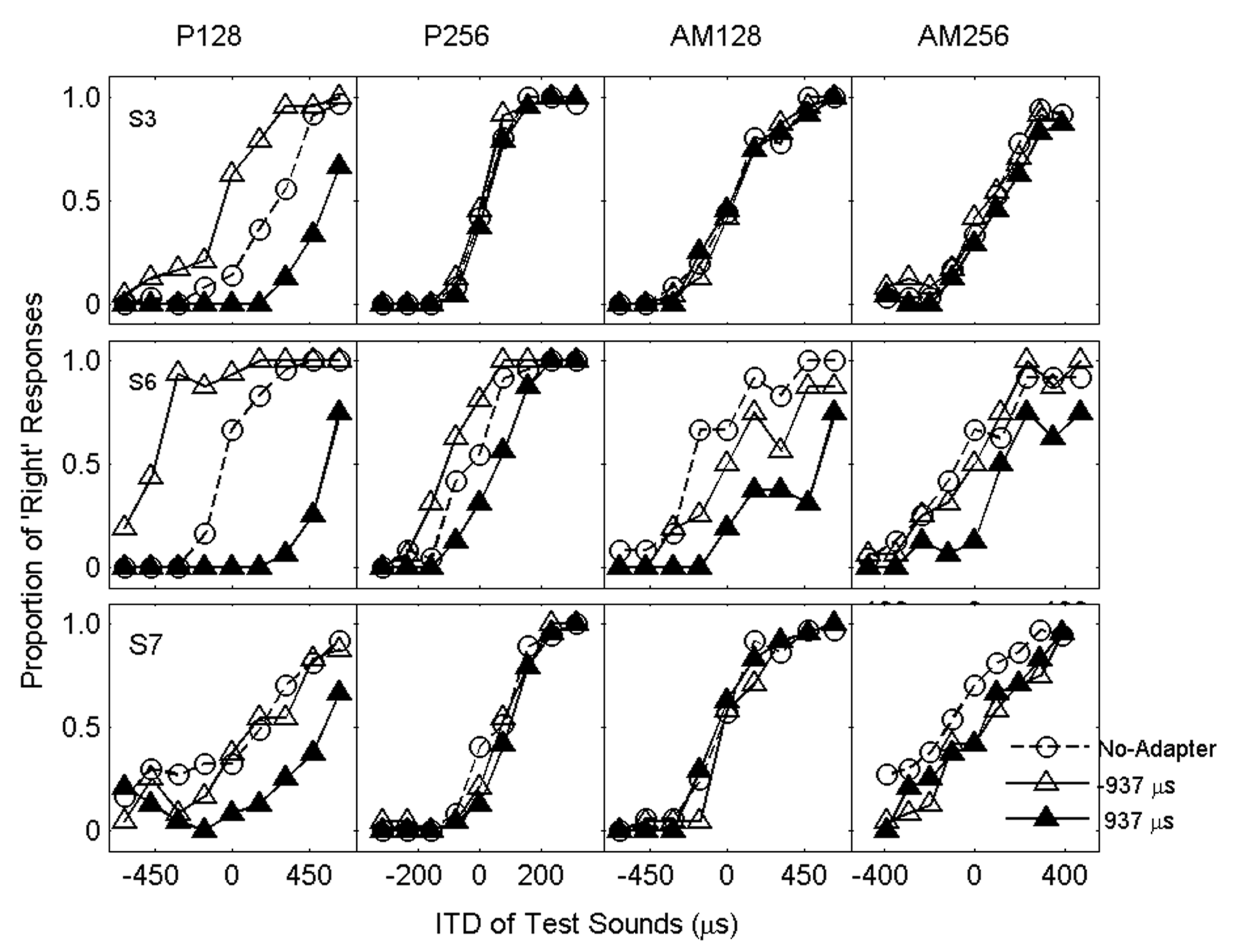

Supplement: Figure S2 — Proportion of “right” responses of each participant as a function of ITD of the test stimuli in the tone adapter condition (128-Hz). See the caption of Figure S1 for details. (TIF) [file pone.0041328.s002.tif]
